# Bone elongation using monolateral external fixation: a practical guide

**DOI:** 10.1007/s11751-015-0236-0

**Published:** 2015-11-25

**Authors:** César Salcedo Cánovas

**Affiliations:** Faculty of Medicine, Unit of Paediatric Orthopaedic Surgery and Traumatology, CSUR – Spanish Reference Unit of the Spanish Health System, University Hospital “Virgen de la Arrixaca”, University of Murcia, Plaza de Fontes, 4. 3º C, Murcia, Spain

**Keywords:** Lengthening, Fixator, Monolateral, Callotasis, Elongation, Guide

## Abstract

In the literature, we can find many articles that describe in detail specific complex procedures related to the limb reconstruction. However, the general information on the biological and mechanical bases of callotasis is out of date, and the surgeons must relate to works dating from the early 1980s. These articles also come from a period in which the callotasis technique was being developed and, therefore, incur in discrepancies depending on the year they were written or the school of the author. This paper provides a general and summarised overview of the theoretical and practical aspects interesting to a surgeon that needs clear information on the bone elongations performed with the help of a monolateral external fixator.

## Objectives

The purpose of this guide is to describe, from a practical point of view, the planning, surgery, management of complications and rehabilitation process when performing bone elongations.

Although many of the considerations made in this paper are applicable to the field of the circular external fixation and even to intramedullary distractors, we will focus on the most common procedure in the Spanish hospitals: the monolateral external fixation.

For the purpose of giving useful information to the reader, we will intentionally omit very repeated aspects in the existing literature and that refer mainly to the history of the development of this kind of procedures, since this does not add too much practical information. Moreover, given the variety of treatments that can be done with modern methods of bone elongation (transport, pseudoarthrosis, malalignments, etc.), we will only talk about simple lengthenings, either by short stature or due to length limb discrepancies.

Although it is common that the trauma specialist is involved in the resolution of limb length differences or hypometrias of very diverse aetiologies, the study of more specific factors such as the analysis of the indications, specific considerations for various diseases, and alternative and/or complementary procedures as chondrodiatasis or correction of associated deformities is outside the scope of this manual, as this would greatly increase the length of the guide and they will be addressed in the future in separate papers.

## Callotasis as bone elongation method

**Fig. 1:**
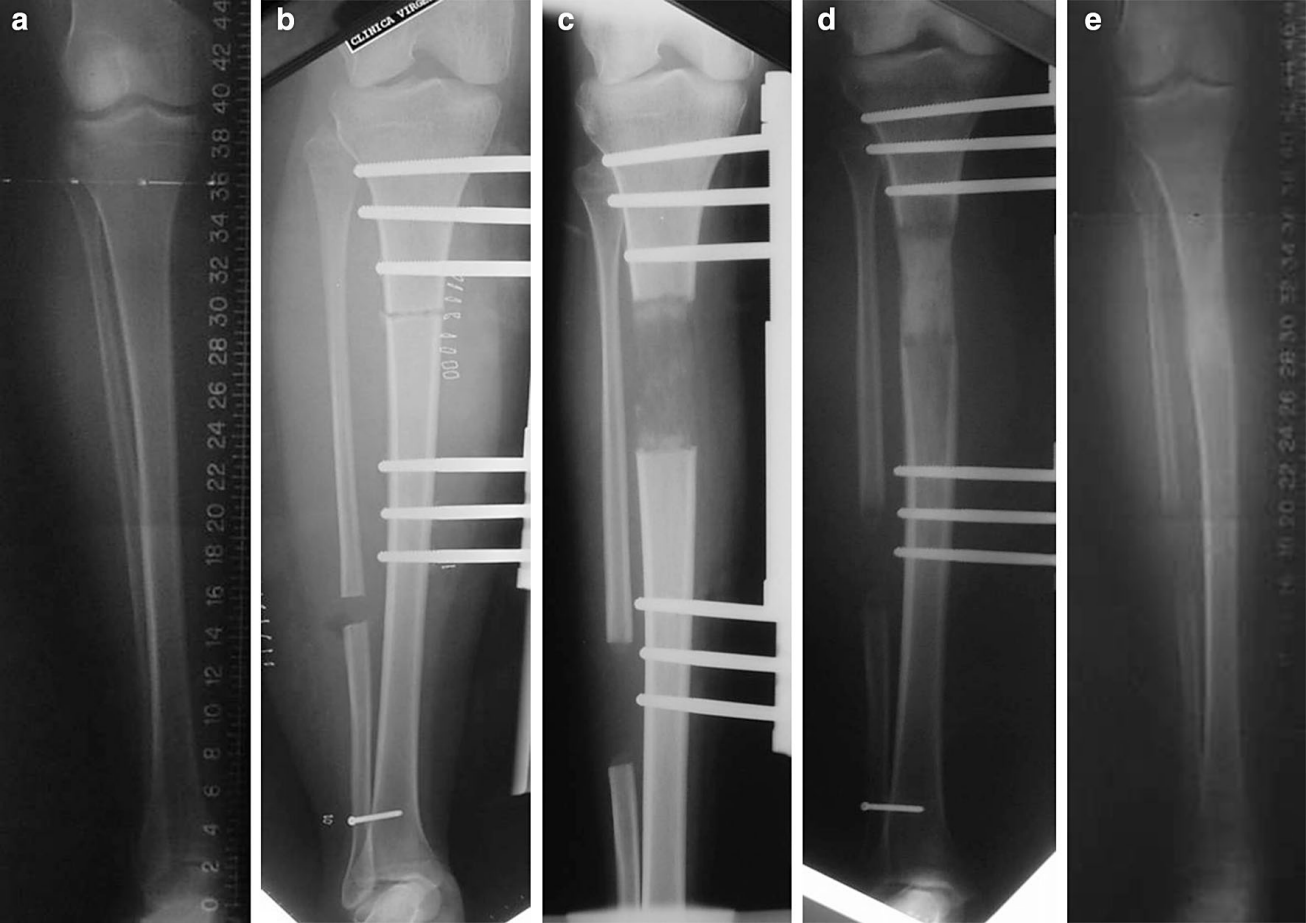
**a** Tibia before lengthening, **b** osteotomy and latency phase, **c** distraction phase, **d** dynamisation phase, **e** tibia after lengthening

Callotasis [1, 2, 3] is the technique of bone elongation developed by the Orthopaedic Institute of Verona (De Bastiani, Aldegheri, Renzi-Brivio and Trivella) based on a set of principles that seek to obtain an indistinguishable bone regeneration of the patient’s healthy bone [4] and minimising the complications of the procedure. The basis of the technique (Fig. 1) are:

- Metaphyseal osteotomy.
- Respect to the surrounding tissues, and especially the periosteum.
- Control of thermal necrosis of the bone.
- Latency period to allow the organisation of the callus.
- Phase of gradual and controlled distraction.
- Neutralisation phase to facilitate callus ossification.
- Dynamisation phase to help the corticalization of the regeneration.

Currently, the callotasis is the most used technique for performing bone elongations due to its proven effectiveness and reproducibility.

## Time to surgery

Although in many cases factors unrelated to the patient or the pathology determine the moment at which the treatment is performed, in order to get the maximum increase in length in patients with short stature, the procedure is recommended to be implemented during adolescent age [5]. Furthermore, making corrections in young patients can avoid the occurrence of compensatory fixed deformities and prevent the deterioration of the patient’s mobility or a deformity that can grow at a higher rate to the potential corrector of the surgery. The optimum age is usually between 7 and 16 [6].

In the case of bilateral lengthenings, we must decide on their order. Both tibias or both femurs can be elongated at the same time, or they can be elongated alternately (one femur and its contralateral tibiae at a time). Currently, the majority of surgeons are choosing to simultaneously act first on tibiae and then to elongate the femurs. Few people use the alternative method since it could lead to asymmetries if the treatment has to be stopped before completion [7].

## Patient selection and planning

Procedural success will depend greatly on the correct indication of it, as well as of the proper planning of all the parameters that configure the deformity.

Anthropometric tables will be used to calculate the percentage ratios between the height and the length of the long bones of the upper and lower extremities measured on radiographs of normal individuals of the age and gender of the patient [5, 6]. Clearly, a complete analysis of the medical history of the patient must be conducted. This will identify possible anaesthetic risks or risks during the surgery or the treatment [6].

For planning purposes, it is essential to have anteroposterior and axial radiographs. Also, the scaled radiographs of the entire loaded lower limb are very useful load to analyse the mechanical axes and to evaluate limb differences [6].

Clinical photographs may be helpful to know the aesthetic effects that the bone deformities make on the external appearance, and for comparative purposes at completion of the treatment.

Calculating limb length discrepancies can be difficult. Of the common clinical methods, it seems that to place blocks of a certain height under the shortened until the iliac crests are balanced is the most simple and reproducible [6].

Limb differences of less than 2 cm are not clinically relevant and do not require surgical treatment [8]. For slightly larger differences, we could envisage the possibility of reducing the length of the longest limb or slow its growth. The callotasis should be reserved for corrections of 5–6 cm or when the patient’s stature is too low. This is because the elongation is a more complex procedure than the control of the length of the contralateral limb [9]. Some authors argue that it is convenient to extend the limb up to 5 mm above the intended length to compensate a collapse that could occur in the dynamisation phase [6].

If there are bone deformities, they must be taken into account when calculating the differences in length, as they can alter their perception. These deformities have to be corrected first in most cases, because they can affect gait and make the process more risky [6] since those deformities might be exacerbated and/or could interfere in the normal function of the limb, difficulting rehabilitation. The versatility of the current external fixation systems makes possible to perform such acts on a single-stage fashion, allowing the correction of angular deformities and discrepancies in length with a lower complication rate.

**Fig. 2:**
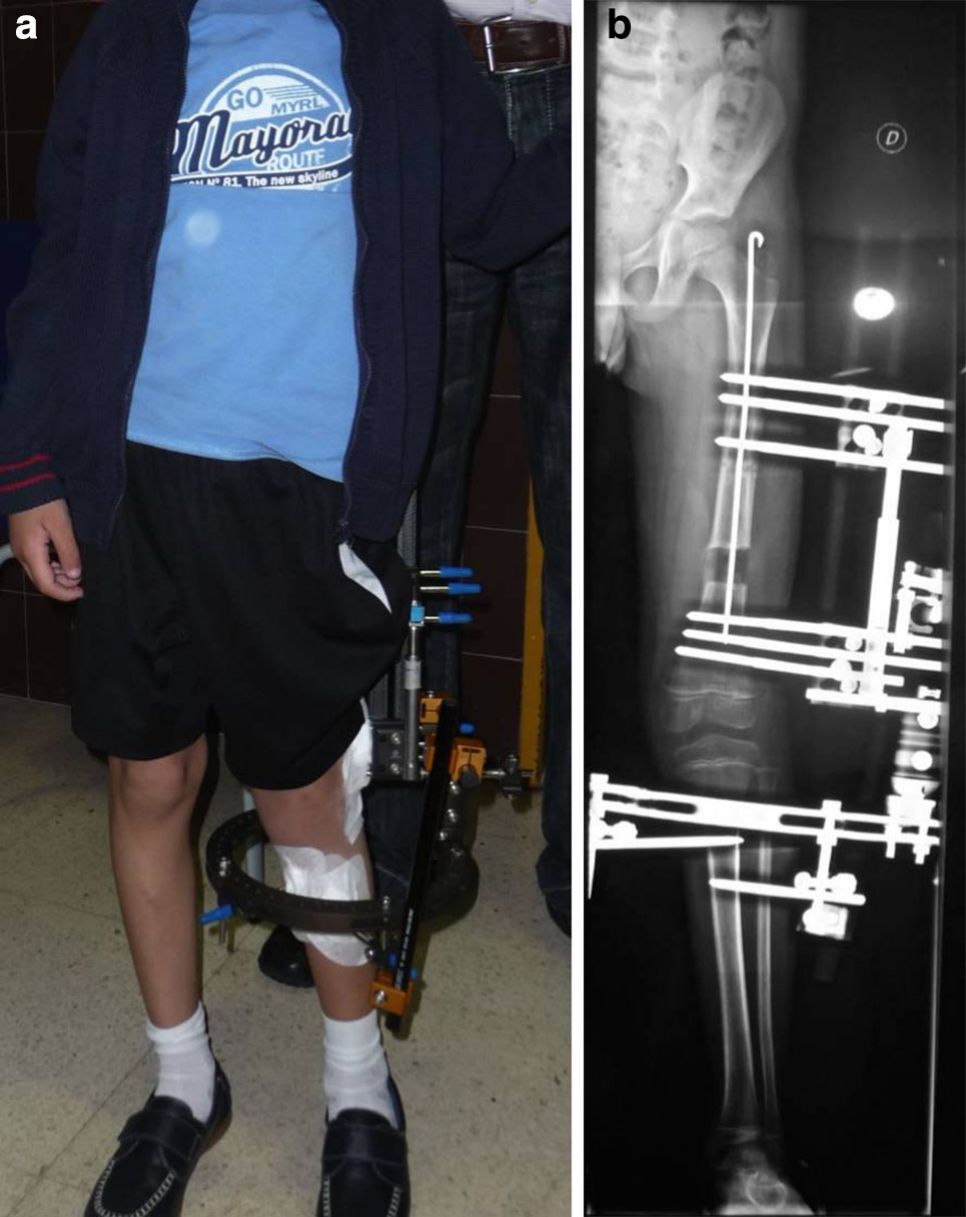
**a** Clinical image of a spanning frame, **b** radiological image of a spanning frame

During lengthening, the soft tissue tension increases. To put a joint that is already unstable under pressure can cause its subluxation. This is very typical in patients with focal femoral deficiencies (congenital short femur) with hip dysplasia or the absence of the anterior cruciate ligament. A child with dysplastic hips could be considered unsuitable for such treatment unless we previously plan a surgical stabilization of the hip as a periacetabular osteotomy or Dega osteotomy. An unstable knee does not prevent the possibility of performing elongations, but it places the patient at risk group [10]. Patients experiencing joint instability might be candidates for the fixator to be positioned as a bridge, blocking the mobility of the joint during the lengthening phase and reducing the risk of subluxation [9] as shown in Fig. 2a, b.

The presence of spasticity is another risk factor. If the forces generated during lengthening act in the same direction as the pattern of spasticity, the joint tension will further increase and the risk of joint damage or subluxation would be also increased [10]. This is why the previous planning with tenotomies, myotomies and fasciotomies is essential for the proper development of the elongation and a comfortable period of rehabilitation.

If the patient has clotting problems, he/she will also be in risk group. Some authors claim that the elongation should not be performed on irradiated bone [10].

Smokers also have a higher risk, as the snuff has negative effects on callus formation [6, 11]. Smoking cessation is advisable prior to embarking into lengthening procedures; besides its negative health effects, it has adverse impact on the treatment the patient is about to start [6].

The psychological profile and the social environment of the patient are very important when tackling a process that can be very long in time. The family must be able to perform the daily maintenance of the fixator and the needs of a patient who will need help with his/her most basic life functions. It is recommended that the patient and family are fully informed of the details of the procedure, and an assessment by psychologists or social workers is recommended. The contact with other patients who have already gone through the process can also be very beneficial [10]. In fact, the most renowned orthopaedic centres opt for multidisciplinary teams to treat these patients. A summary of the factors to be analysed is given in Table 1.

**Table 1:** Factors to consider in elongations

Angulation
Torsion
Osteoporosis
Joint instability
Scoliosis
Muscular weakness
Neurological injuries
Musculotendinous contractures
Coverage or quality of the soft parts
Infection
Collaboration of the patient and his/her family

## Desirable features in a fixator for elongations

The first decision that a surgeon must make when choosing a system of external fixation to elongate a limb is to choose a monolateral fixator or circular one. Each of them has a number of advantages and disadvantages that must be know to fit the needs of each case. Circular fixators are usually much more versatile, and their use is indicated for severe bone deformities or in complex anatomical locations, such as the foot. In return, the technical management of them is more complicated; they are more uncomfortable for the patient and require a higher level of training by the surgeon. When treating a simple lengthening as the ones we consider in this guide, the transfixing systems have a higher rate of associated risks and do not offer better clinical results than those obtained with quality unilateral fixators [3, 11]. However, the surgeon’s personal preferences play an important role in making the final decision.

**Fig. 3:**
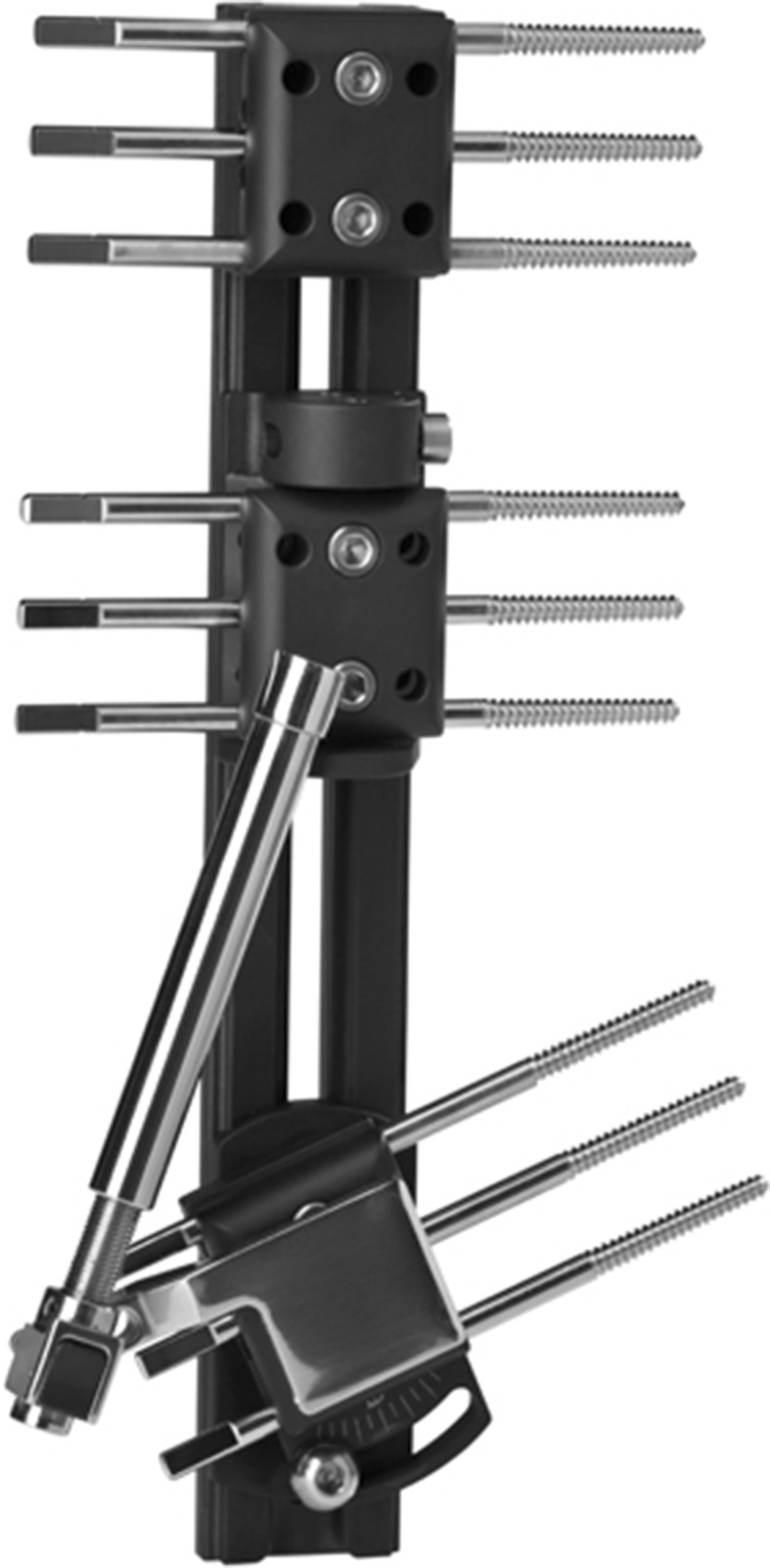
Monolateral fixator designed for lengthenings (LRS ADV, Orthofix SRL, Verona, Italy)

Assuming we choose a monolateral system, it would be interesting to use a fixator with a single bar that controls the lateral and anteroposterior bending and the torsional forces (Fig. 3). It should allow for controlled distraction, the application of compression at will and the transmission of dynamically axial load once the callus has been formed [12, 11].

The stability of the assembly is a crucial factor for a successful outcome, and it is determined by the design of the fixator, the screws and the spatial arrangement in which the surgeon applies the assembly. The lack of rigidity of an external fixation system favours the formation of a cartilaginous callus that finds it difficult to turn into bone. The clinical consequence would be a delay in the consolidation and even the appearance of pseudoarthrosis [3]. On the other hand, too much rigidity in the later stages of the treatment may also hinder the consolidation of the bone regeneration. That is why the optimum fixator must allow the surgeon to adjust the degree of axial strength at his/her discretion.

Also, it is equally important that it is lightweight and with an ergonomic design that allows the patient to develop normal functions [12]. This will result in greater comfort for the patient, but also in a higher level of treatment success, since rehabilitation and ambulation on the limb will be easier to perform.

## Desirable features for fixation half-pins

The bone screws are a key part of the fixation system, as they are responsible for transmitting the forces between bone and the external tutor. Therefore, the design must be configured so that it can support forces of tension, compression and bending while avoiding problems of biocompatibility.

The most used material for the manufacture of the screws is stainless steel, which excellently supports the loading forces and has shown a great experience in clinical use.

The half-pin diameter must be as big as possible to prevent deformation. Screw bending generates forces at the junction with the bone which may lead to the development of osteolysis and its loosening. However, if the diameter is too big, this can weaken the bone to the point of breaking it [13]. As a general rule, the diameter of the screw must not be over one-third of the diameter of the bone in which it is fixed [14].

The design of the threaded section of the half-pin may be tapered or cylindrical. The tapered ones remove new bone in every turn as they are inserted. Thus, the osteolysis is reduced and a radial preload is generated, which increase fixation. The conical shape is also consistent with the fact that most of the forces that the screw withstands in a monolateral fixator are produced in the closest cortical to the assembly. A conical half-pin also has the advantage of being easier to remove. In turn, its major drawback is the inability to make adjustments in its position if we need to go back a few turns, since it would be loosen.

The thread design of the external fixation screws must be symmetrical, as they must withstand forces from all directions [13].

Although there are self-drilling half-pin, in elongations or long-term procedures its use is not recommended. Drilling used during standard technique reduces the temperature increase that occurs when inserting the screw and prevents possible bone splintering when it reaches the second cortex. In addition, the self-drilling screws generate more osteolysis, especially when they reach the second cortex (harder than the canal), and it takes several turns to get the thread engage the cortex, damaging the thread that had already being done in the first cortical area and in the medullar canal. The drill bit must be selected according to the diameter of the pin, the shape of the thread and bone quality.

But perhaps the most decisive factor in relation to the fixation of the half-pins is the use of hydroxyapatite coating. All metal screws are progressively loosened over time, while the ones coated with hydroxyapatite have shown an increase in its grip thanks to the osseointegration. A better grip reduces the mobility of the screws, and this reduces the loosening, the inflammation and the possibilities of infection. The use of hydroxyapatite-coated half-pins is very important when conducting a long-term treatment such as bone elongation [15]. On the other hand, its good osseointegration complicates its extraction and their removal has to be done in a protocolled manner and with appropriate tools. It is not uncommon that some patients require anaesthesia.

## Bone screw selection

The thread length shall be such that it will allow two or three threads to protrude through the second cortex of the bone and that about 5 mm of it will lie outside the first cortex, ensuring good fixation [1, 13]. It is important that the smooth part of the half-pin does not penetrate into the bone, since it will surely cause loosening, mobility and infection. Similarly, it is not recommended that the thread goes through the skin, since it can be a too direct line of communication between the outside and the bone, being a constant risk.

The total length of the screw will depend on the thickness of the soft tissues and the position of the fixator [6]. However, new systems allow for the use of very long half-pins that can be cut to the proper size afterwards.

**Fig. 4:**
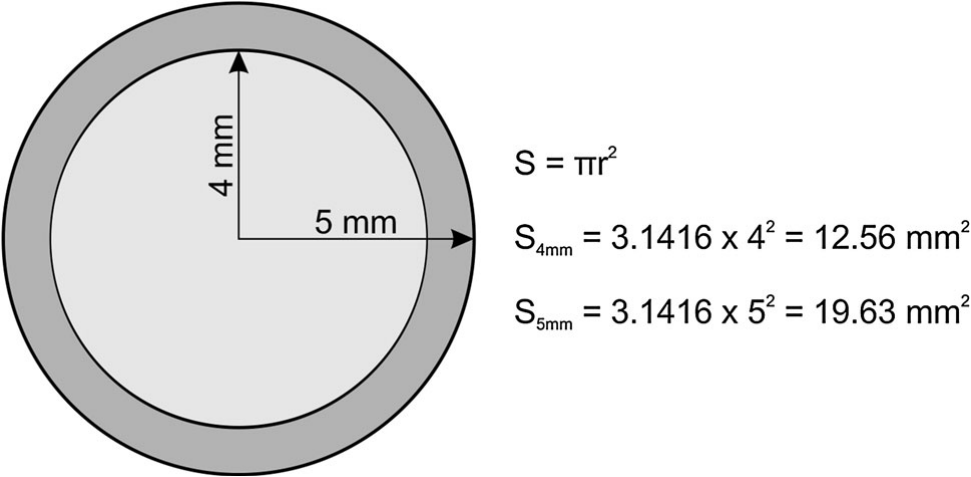
Different screw sections in 4- and 5-mm bone screws

The diameter of the half-pins affects the rigidity of the assembly. For example, when it changes from 4 to 5 mm, the sectional area increases to 50 %. This increase in the section also acts on the moment of inertia, decreasing the forces to the level of the screw–bone interface, and prevents the loosening of the bone anchor (Fig. 4).

Although in the past we distinguished between cortical and cancellous pins, almost all modern systems of fixation have chosen a universal thread, a thinner one that increases the contact surface between bone and bolt, regardless of the trabecular structure of the region to be treated.

## Screw insertion technique

Insertion technique must be careful to avoid thermal bone necrosis and subsequent infection [6, 11]. Poor insertion technique in lengthy treatments often results in pin failure [11]. The duration of the assemblies when we implement an elongation depends on the rigour in the implantation technique of the bone screws. Still, we can never be sure that a half-pin is not going to become intolerant or infected.

**Fig. 5:**
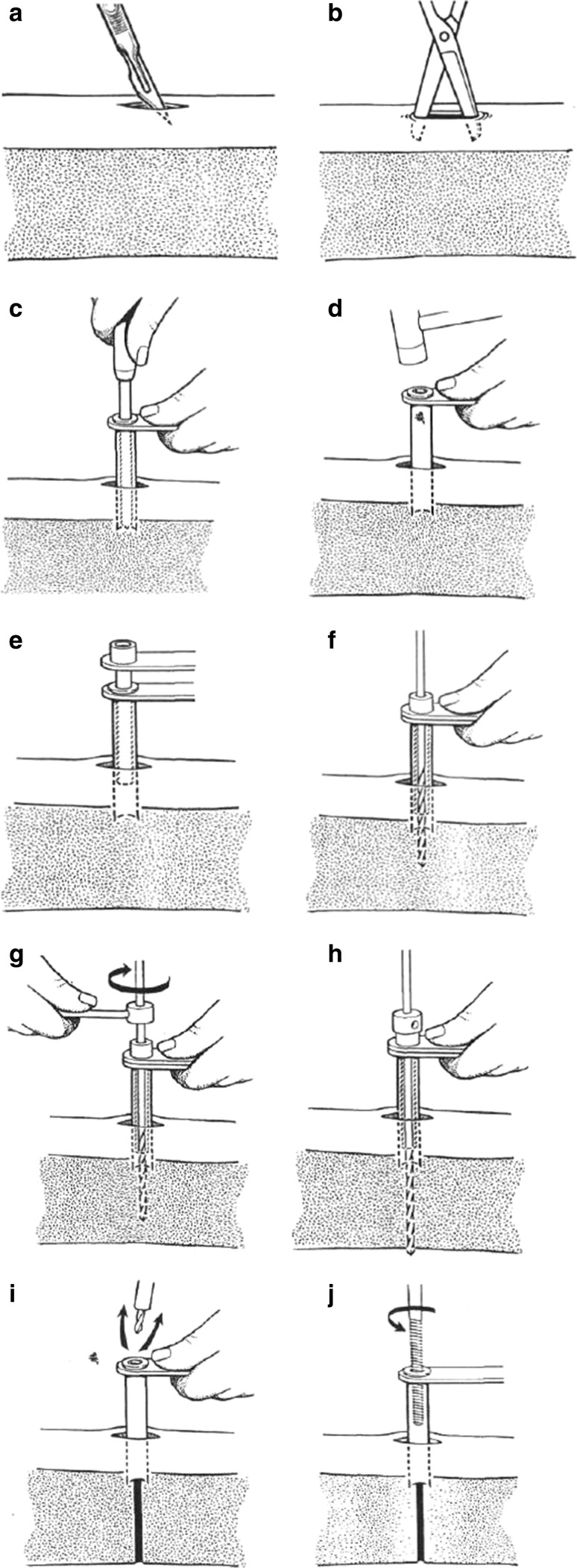
**a** Skin incision, **b** blunt dissection, **c** trocar palpation through screw guide, **d** impaction of the screw guide, **e** drill guide, **f** drill first cortex, **g** use of drill stop, **h** drill second cortex, **i** removal of drill guide and drill, **j** insertion of bone screw

The recommended surgical technique for the insertion of bone screws is as follows [6] (Fig. 5):

- Small longitudinal incision and adequate soft tissue dissection.
- Push the screw guide and the trocar to the bone.
- Feel the bone surface with the trocar, ensuring the proper positioning.
- Remove the trocar.
- Insert the drill guide through the screw guide.
- Use a clean sharp drill with the appropriate diameter.
- Drill at low speed (below 600 rpm), avoiding the bone heating and with constant pressure. The use of a drill stopper is recommended.
- Removal of the drill and the guide.
- Some authors recommend irrigating with saline.
- Use templates for correct positioning of the pins; if we do not place the screws in parallel, it will lead the fixator to apply excessive forces on them that increase the risk of osteolisis [11].
- Clean the drill between applications.
- Check the range of joint mobility and release the soft tissues if necessary.
- Apply non-adherent absorbent dressings.

## General technique to apply a monolateral fixator in lengthenings

### Femur

The fixator is located on the lateral aspect of the femur. Some authors consider that the body has to be placed slightly anterior to avoid inconveniencing the patient when the leg falls in external rotation during sleep [6]. We recommend using six half-pins (three per clamp). This is because the stability of the assembly is higher than with four screws and because the failure of a single fixation element will allow its removal without compromising the whole procedure [11].

Position of the first screw will be the most distal in the proximal clamp and will be implanted at about the level of the lesser trochanter and perpendicularly to the diaphysis [1, 2]. The new fixators allow for a preliminary fixation with Kirschner wires before implanting the screws, which facilitates the repositioning of the frame without damaging the bone by successive drillings.

**Fig. 6:**
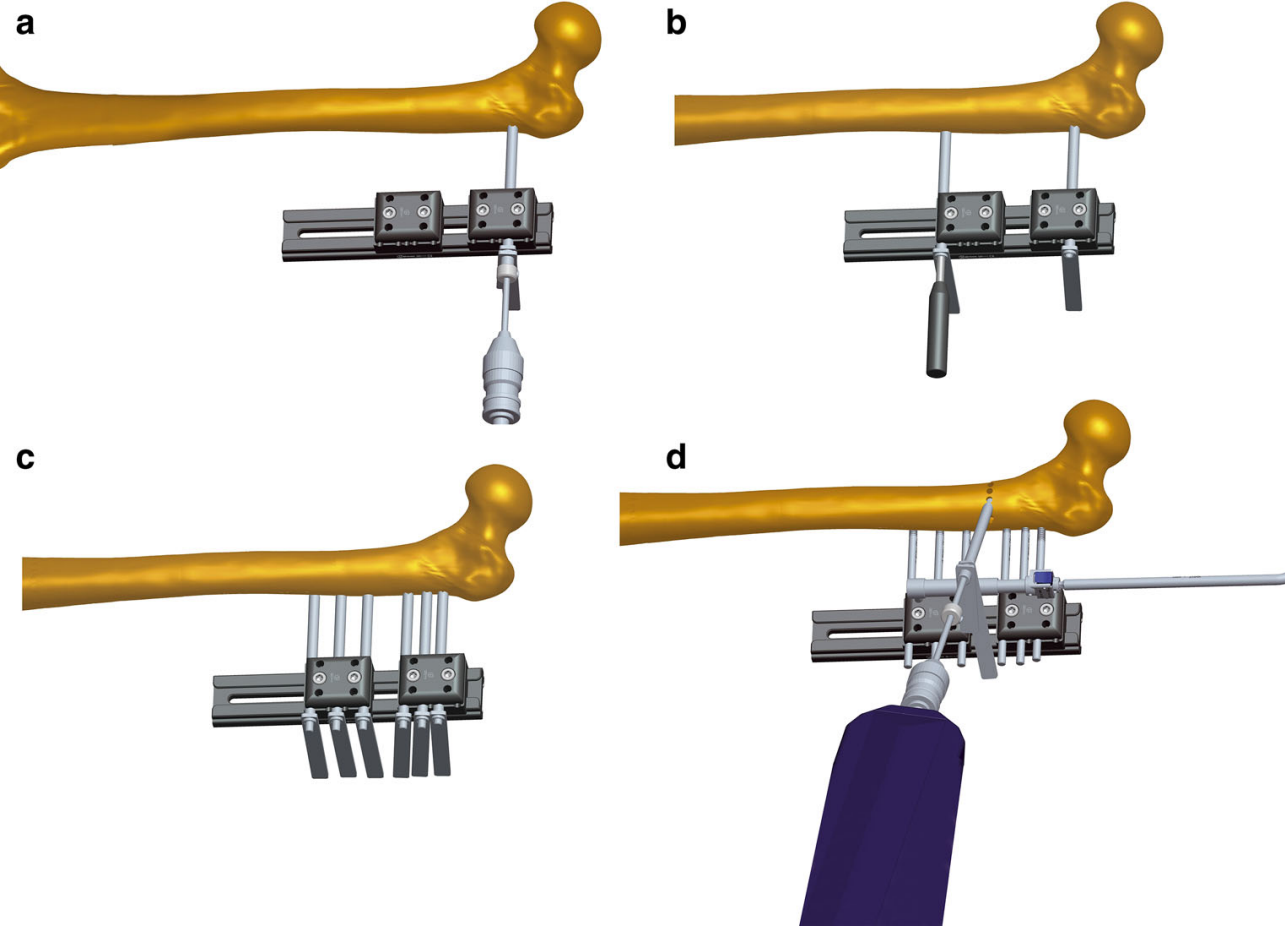
**a** Insertion of the most proximal screw, **b** insertion of the most distal screw, **c** insertion of the rest of the screws, **d** tension osteotomy

**Fig. 7:**
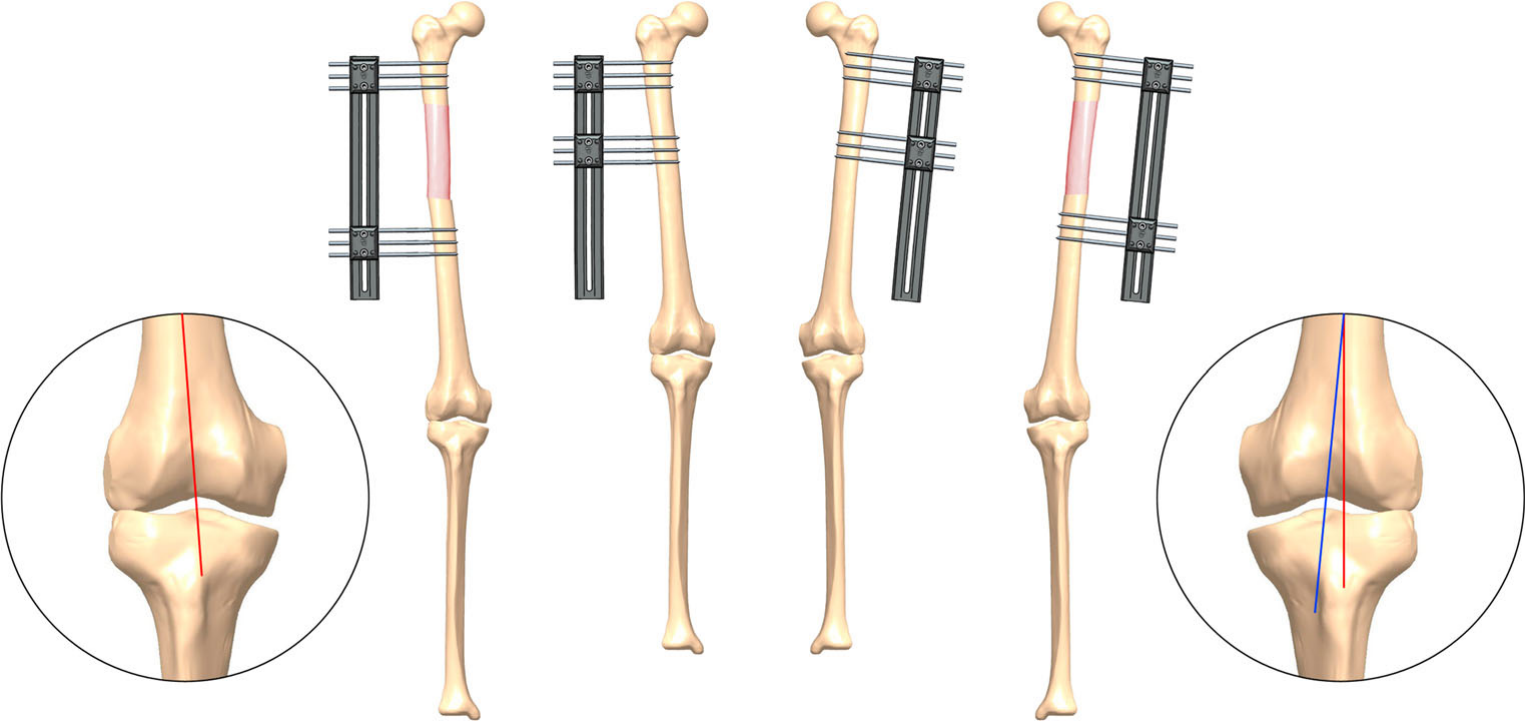
Lengthening along the mechanical axis (*left*) and the anatomical axis (*right*)

The decision to place the body of the fixator with respect to the anatomical or mechanical axis of the bone to be lengthened is important, and it will depend on the situation of the first screw (unless articulated clamps are used). For some authors, it is important that the longitudinal axis of the fixator is parallel to the femoral diaphysis, following the anatomical axis of the femoral bone [1, 2]. However, when the femoral lengthenings exceed 7–9 cm, we can see an increase in femoral valgus above 7°. If the elongation axis is not parallel to the mechanical axis, a minor deformity will occur during the gradual increase in length. An axis of elongation parallel to the anatomical axis of the femur shall move the knee medially and force the mechanical axis in the lateral direction, resulting in a valgus deformity of the knee. Per each centimetre of elongation along the anatomical axis, the mechanical axis is laterally displaced 1 mm. For this reason, other authors consider that the body of the fixator has to be placed in parallel with the mechanical axis, preventing the valgus and the medial translation during the lengthening, [6] as shown in Fig. 6. Nevertheless, we also have to take into consideration that placing the body of the fixator parallel to the mechanical axis will result in having more stress on the pin bone interface since the screws will not be inserted at right angles into the bone. Also, the frame will not be parallel to the limb (Fig. 7).

Secondly, we will look for the best location for the most distal half-pin of the fixator. Once we have placed the first two fixation elements, the placement of the remaining ones will be routine and guided by the clamps of the system [1, 2].

Any tension in the soft tissues around the screws should be released, and we must check that the range of motion of the limb is adequate [1, 2]. We should try to have a knee flexion of at least 90° [6].

It is recommended to assess the condition of the soft tissues. When treating elongations over 5 cm, we can choose to relax or lengthen tendons that could strengthen unwanted deviations. Thus, we can make tenotomies of the adductor medius tendon and the tensor of the fascia lata, and in major bone distractions where there may be a tendency to flexion the hip, we will consider adding gestures as tenotomise the anterior rectus tendon at its origin of the anterior–inferior iliac spine.

**Fig. 8:**
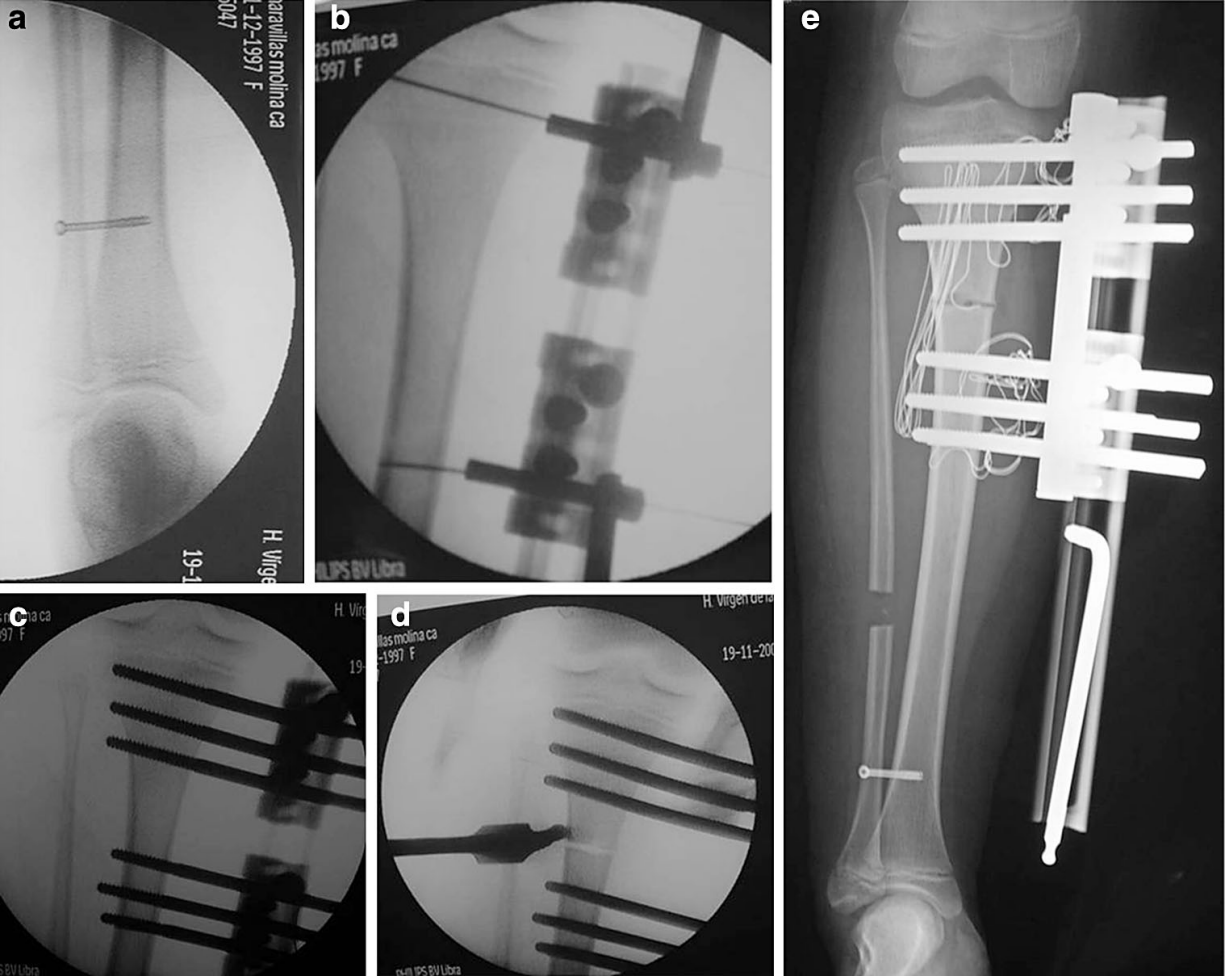
**a** Syndesmotic screw, **b** locations for the most proximal and distal screws, **c** insertion of the screws, **d** osteotomy, **e** end of surgery

### Tibia

The medial positioning of the fixator would give high stability, although it would be uncomfortable for the patient to walk, because the assembly would interfere with the contralateral tibia. To avoid this, we often opt for an anteromedial application, which provides sufficient stability and prevents the patient’s discomfort (Fig. 8). Some authors recommend the anterior placement, stating that the aforementioned configurations favour valgus deformities [6]. Combining an anterior placement with the use of a T-Garches swivel clamp, we can correct malalignments during the treatment without additional surgeries [5].

As with the femur, using six half-pins (three per clamp) is recommended. This is because the stability of the assembly is higher than with four pins and because the failure of a single fixation element would allow its removal without compromising the procedure [11].

There is no controversy about the tibia, if the fixator must be implanted in parallel to the anatomical axis or the mechanical axis, since both are coincident [6].

The first screw will be the most proximal one and will be placed as high as possible without invading the joint capsule. The joint line can be used as a reference to place it parallel to it. Subsequently, we proceed to implant the most distal screw of the frame. Once these two elements have been placed, the remaining ones will be guided by the heads of the fixator and their implantation will be routine.

## Osteotomy

The objective is to interrupt the bone continuity so that the optimum local conditions for callus osteogenic proliferation [3] are obtained. Although it would be desirable to respect the endosteum and intramedullary blood supply as Ilizarov advocates, in the clinical practice it seems inevitable to damage it [6, 11]. However, several studies establish that its recovery is quick and that its importance in ossification is well below the ossification of the periosteum [16].

**Fig. 9:**
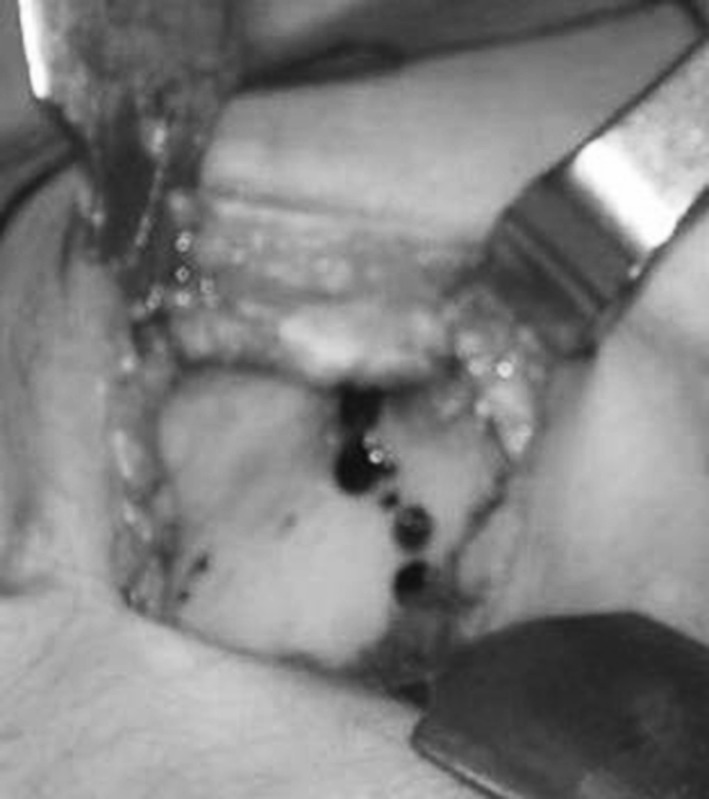
Osteotomy

In the femur, the osteotomy is performed just distal to the insertion of the iliopsoas; in the tibia, distal to the insertion of the patellar tendon; and in the humerus, distal to the insertion of the deltoid [1, 2]. It is recommended to do the osteotomy in the metaphyseal region, because in that area the bone has a larger transversal section, the cancellous bone is more abundant, and the periosteum is thicker [3]. In short, it is the region with the highest osteogenic potential.

The approach has to be minimally invasive, preserving the coverage of soft parts and the blood supply to the area. In the femur, the approach is anterior, acceding between the sartorius muscle and the tensor of the fascia lata, crossing the fibres of the vastus intermedius and the rectus femoris. A simple skin incision showing the anterior side is enough in the tibia, and to implement the humeral osteotomy, we use the space between the long head of the biceps and the deltoid and brachial [1] muscles [2].

We more and more tend to make the osteotomy percutaneously, since doing it this way we give more protection to the coverage of the soft parts, we do not have too much periosteal stripping, and we facilitate the osteogenesis. Thus, percutaneous perforation techniques have been proposed through guided drill after which we link those orifices through osteotome by practicing a low-intensity osteotomy (Fig. 9). For other authors, the use of the Gigli saw is usual. If you choose this option, the Gigli saw must be introduced at the beginning of the surgery, prior to the application of the external fixator (especially if it is circular).

Some authors defend that it is advisable to apply some axial distraction over the fixator bolts before implementing the osteotomy. This would facilitate the rupture of the posterior cortical area without drilling it directly. This avoids damages to the periosteum due to blind drillings [2, 6].

The periosteum is sectioned longitudinally and must be risen gently, practicing the osteotomy under it and preventing damage to retain its osteogenic potential. To do so, we use a 4.8-mm drill bit through a drill guide. Using a drill stopper that restricts its projection to a centimetre beyond the guide will prevent damages to the periosteum of the opposite end of the bone [1, 2, 6]. We implement a series of holes aligned in the accessible part of the bone, and then, we link them with an osteotome.

Another option is to perform the osteotomy in a completely percutaneous fashion. Nowadays, many surgeons attempt to limit the incision just large enough to insert a drill guide to perform the osteotomy through arch movements, minimising the damage made to the soft tissues and blood supply to the bone.

The posterior cortex should be broken by osteoclasis thanks to the tension previously applied, and the surgeon must verify that the osteotomy is completed by separating the ends of it using the same distraction capacity of the fixator. Then, the ends are returned to their original position.

The surgery is completed by suturing the periosteum, whenever possible, and the skin. In selected cases, it could be beneficial to leave a temporary drain although we do not do it routinely, since we do percutaneous osteotomies and they rarely cause compartment syndrome.

## Associated acts

In the tibial elongations, 1 or 2 cm of the fibula is resected before implementing the tibial osteotomy. If we remove a good portion of the bone and the periosteum, we can avoid the risk of premature consolidation [1, 2, 3, 5, 6]. But if we are only going to perform an axial elongation, it is also possible to implement a simple fibular osteotomy without resection.

Fixing the distal fibula to the tibia prevents the rise of the malleolus and the subluxation of the ankle. Tibial elongations greater than 2–3 cm without fixation of the joint can cause instability in valgus of the ankle, impacting negatively on the progress and stability of the joint. Therefore, we advocate the temporary fixation of the distal tibiofibular by means of a 4- to 4.5-mm cannulated screw. This screw will be removed 6 months after removing the fixator [5].

Immobilizing the proximal tibiofibular joint is not a widespread option.

When lengthening tibias, we can also practice a percutaneous tenotomy of the Achilles tendon in the same surgical act as the osteotomy. Thus, the subsequent emergence of equinus deformity is prevented. After surgery, we immobilize the ankle in plantigrade position with a splint, although physiotherapy also plays a key role in reducing the stresses produced during the elongation [5, 10]. Unfortunately, the indications to include the foot in the fixator during the tibial lengthening are not entirely clear. It is essential in severe shortenings greater than 5 cm associated with bone dysplasias of fibular hemimelia type with tibiofibular–talar joint in “ball and socket”, because the equinus is usual. In small shortenings with normal ankle mobility, it is not required. If there is a previous equinus or a relative lack of dorsiflexion, then it is necessary to perform a lengthening of the gastrocnemius fascia or the Achilles tendon and use a fixation on the foot in a slight dorsiflexion. This fixation can be removed during the healing phase provided that it is not associated with a knee flexion contracture.

Similarly, in the femoral lengthenings we can practice a percutaneous release of the gracilis, sartorius and rectus femoris. Thus, the chances of deformities in the hip and/or knee due to the increased tension of the soft parts [10] are reduced. We usually perform femoral releases in some pathologies such as congenital short femur on in massive lengthenings where soft tissues will not stretch at the same rate of the bone and their tension can create joint contractures. In general, lengthenings over 5 cm could be considered for soft tissue release.

## Latency phase

After surgery, the patient may begin to perform partial load from the first day (if the fixator is rigid enough). The assembly will stabilize the osteotomy to allow the haematoma and the callus [1, 2, 5, 11].

The latency phase has two objectives [3]:

- To facilitate the healing of the surgical wounds affecting the pericortical and periosteum vasculature.
- To facilitate the cell proliferation forming the bone bridge, which stabilizes the two bone segments.

The osteogenic capacity of callus is much higher if an adequate revascularization is allowed. Otherwise, it is highly possible that the ossification will be eroded by local ischaemia [3].

Although the duration of this phase varies depending on the age of the patient, the type of disease and many other factors, usually it is between 7 and 10 days after surgery. It seeks a balance between good callus formation and the risk of early consolidation of the osteotomy.

## Distraction phase

Once radiographically observed that the callus begins to form, the osteotomy begins to distract at a rate of 0.25 mm every 6 h. If there is pain or muscle spasms, the elongation pace may be slower [1].

A week later, another radiograph is taken to check the correct separation of the bone ends, and then, monthly follow-ups are made. If there are signs of poor callus formation, the rate of distraction can be reduced. It is even possible to temporarily reduce the length of the bone if vascular or nerve problems [1] are detected. Oppositely, if we observe a too high ossification that indicates a risk of premature consolidation, the rate of elongation may be temporarily increased.

## Neutralisation phase

Once we have obtained the desired length, the fixator is locked to stabilize the bone regeneration. At this time, the total load weight on the limb is highly desirable in order to achieve maturation and ossification of the callus [11].

## Dynamisation phase

Finally, once the callus is mature enough, we place the fixator in dynamic mode, allowing gradual axial load. In a first phase, silicone bearings or similar elements will be used to prevent the collapse of the regeneration and, later, we will use free dynamisation (the fixator controls the torsion and the bending, leaving axial load at the expense of the bone) [11]. The moment of the dynamisation is difficult to be determined with precision, but the surgeon can estimate it by searching for some initial corticalization on the X-rays.

The dynamisation increases bone thickness facilitating the corticalization and prevents fractures or malunion after removing the fixation device [3, 16].

The use of excessively rigid fixation systems can cause delays in bone consolidation, while the models that allow for some movement of the fracture show a proliferative callus formation. It is also true that, above a certain level of mobility, the callus formation is inhibited [16]. The controlled axial load improves bone healing. Conversely, the movement of the bone in other planes causes shearing, bending or torsional forces inhibiting the bone formation. The ideal fixator should be able to control the movements this way [11].

Furthermore, allowing the bone to support a great part of the body weight eliminates stress of the fixator half-pins, which reduces the chances of osteolysis around them [3, 11].

## Removal

When the regenerated bone corticalization is confirmed, we remove the fixator. Some centres recommend leaving the bone screws in place for 3 or 4 days in order to be able to reposition the fixator in case of length loss or fracture [1].

 The callus rigidity is the most important mechanical parameter when considering the healing of the regenerate. The removal moment remains unclear nowadays, as there is a re-fracture in between 3 and 11 % of cases. The most used method in the daily practice to observe the consolidation of the regenerate is a simple radiography, which only provides qualitative information not directly related to the mechanical properties of the bone. If we successfully measure the rigidity quantitatively, we may be close to solving this difficulty. In this sense, studies have been published concluding that the consolidation of the tibial fractures is optimal for the removal of the fixator when we reach a rigidity of 15 Nm/degree (range 8.5–20) in the sagittal plane. It has been found that the incidence of refractures is lower in the fractures subject to rigidity control and that in these fractures the removal of the tutor is implemented 2–3 weeks before than in the fractures subject only to clinical and radiological control

Sometimes, it may be desirable to give additional support to the bone with a functional splint or other temporary immobilization method [10].

## Healing rate

The healing rate is an expression of the number of days of treatment required for the consolidation of a centimetre in an elongation. This is obtained by dividing the total treatment time (in days) by the elongation achieved (in cm) [1]. Obviously, the new bone has to present the mechanical properties that characterise a healing (loading stand without pain or fractures or deviations).

The rate depends on the patient, the elongated bone, age and pathology. It seems to be relatively independent of the length of the regenerated [5].

Most of the published studies show that the humerus is the bone with the lowest healing rate, while the tibia is the bone that takes longer to consolidate after its elongation. The femur is usually between the two of them.

The origin of the shortening of a limb is very important as to the quality of the regenerated. The clearest example is shown in the bone of an achondroplastic patient with a great ossification potential when it is subject to an elongation, while the ability of osteogenesis in a congenital short femur or a fibula hemimelia may be limited due to their dysplastic origin.

## Complications

The bone elongations are procedures that, due to their long duration in time, are likely to generate a large number of complications. Their proper identification and handling by the surgeon will determine, to a large extent, the future success of the treatment. Table 2 shows some of the most common complications and the solutions that are most frequently applied.

**Table 2:** Complications in elongations and their solutions

Type	Complication	Solutions
Bone	Early fusion of the osteotomy [2]	To increase the distraction rate [11] Non-surgical anesthetized handling [2, 6] To re-implement the osteotomy [2]
	Fusion of the fibular osteotomy [2, 5, 6]	Broader resection of the bone and the periosteum [2, 5, 6]
	Fractures after removing the fixator [2]	Re-application of the fixator [2, 6]
	Osteomyelitis [6]	See “infection” section
	Septic arthritis [6]	See “infection” section
	Insufficient osteogenesis [5]	To respect the periosteum during surgery [6] Reduction in the distraction rate [6] Compression and then distraction again [6] Application of the graft [5] Electro-stimulation
	Non-consolidation [5]	Graft Internal fixation Electro-stimulation
	Axial deviation [5, 6]	Correction by using the articulated heads [5, 6] Corrective osteotomy [5, 6]
Muscle and joint	Movement range loss [6]	Physiotherapy [6] Soft parts release [6]
	Sub-luxation [6]	If there is joint laxity, we can prevent it with a bridge assembly during the elongation In femoral lengthenings, we implement a percutaneous release of the adductor longus, gracilis, straight head of the rectus femoris, sartorius and fascia lata [6, 11] In tibial lengthenings, fixation of the fibular head
	Clubfoot [2]	Percutaneous elongation of the Achilles tendon and plantigrade fixation of the foot with a splint [2]
	Patella alta [5]	Elongation of the quadriceps tendon and rehabilitation^5^
	Patella baja [5]	Transposition of the tibial apophysis 6 months after completion of the lengthening [5]
Neurological	Neurological damage [6]	We must avoid ipsilateral femur and tibia elongations to prevent this kind of problems [11] The distraction rate is reduced, and even the limb is shortened [6]
Vascular	Bleeding and compartment syndrome [6]	
	Aneurysm [6]	
	Hypertension [6]	
	Deep vein thrombosis or pulmonary embolism [6]	
Bolts	Infection [2]	See “infection” section
	Instability due to osteolysis [5]	It is prevented with an appropriate surgical technique and the use of bolts with HA Removal of the bolt and substitution, if necessary
	Bending or breakage of the bolts [16]	Removal of the bolt and substitution, if necessary
Wound	Abscess of soft parts [5]	Curettage [5]
	Pain [6]	Analgesics The distraction rate is reduced and even the limb is shortened [6]
	Haematoma [6]	
	Dehiscence [6]	
	Infection	Level I: cleaning and intensive massage [6] Level II: oral antibiotics [6] Level III: intravenous antibiotics or in the insertion area of the bolts [6] Level IV: removal of the bolt and antibiotherapy [6] Level V: removal of the bolt and surgery to control the infection of the bone [6] Level VI: no response to treatment (chronic osteomyelitis) [6]

## Postoperative management and physiotherapy

A previous assessment must be implemented before surgery. The joint stability and the range of motion of the knee, hip and ankle will be checked. It is also important to analyse the spine to locate any compensatory deformities. We must check that the sensory is normal, as well as reflexes and strength of the limb [10].

In the immediate postoperative period, pain is controlled through continuous infusion by an epidural catheter or an analgesia machine controlled by the patient. Later, oral analgesics will be provided if necessary [10]. It is important that the patient suffers little pain, especially if we can anticipate that he/she will be subjected to subsequent elongations. Thus, negative memories about the surgery [6] are reduced. Nevertheless, some drugs such as diclofenac-derived NSAIDs are not recommended since they can inhibit new bone formation. Other commonly used analgesics have not shown adverse effects related to osteogenesis.

The lengthening causes an increase in the tension of the soft tissues, and this tension increases with the length. There are some common patterns of muscle spasm that can occur in bone elongations procedures [10]:

- Tibial lengthenings: the equinus deformity is common. There may also be knee flexion, although it is less frequent. These deformities are caused by the tension generated on the gastrocnemius, which crosses both joints.
- Femoral lengthenings: can cause flexion and adduction at the hip, as loss of flexion or extension at the knee. The flexion deformity of the knee is more dangerous because it can lead to a subluxation.
- Simultaneous lengthenings in femur and tibia: deformity may appear in flexion and abduction of the hip, clubfoot and limited in the motion of the knee.

There are two ways to address musculotendinous contractures: either preventing them during surgery by releasing soft tissues or using active physiotherapy throughout treatment to prevent them. Perhaps, the most interesting strategy is to combine both approaches depending on the characteristics of each case [10].

Muscle tension reaches its top at the end of the distraction period. During the neutralisation, it begins to decline, facilitating the joint movements.

The caring of the screws now belongs to the patients and their families after a proper instruction. The control of the distractor is also at their responsibility [6].

The check-ups are carried out weekly or fortnightly during the early parts of treatment. Then, the check-ups will be monthly until the removal of the fixator [6].

## Bone screw care

The patient must receive adequate training on how to properly clean screw insertion sites and the external frame [6]. Although the nursing protocol depends on the preferences of the hospital or the surgeon, we can provide a number of general principles that are generally accepted. A possible approach would be as follows:

- The patient must wash his/her hands thoroughly and dry them with disposable paper.
- The patient must massage the skin around the half-pins trying to drain the fluids or the dirt to the surface.
- A cotton bud is soaked with a cleaning solution determined by the surgeon. The solution is applied pin by pin in circular motions from the inside out. Any crust that has been formed is removed and a different bud will be used for each screw, avoiding possible microorganisms to move between the different insertion points. After completing each pin, the patient will use a new piece of cotton to dry the area.
- The full length of the screw must also be cleaned with a piece of gauze, which must be changed between screws.
- The patient must roll an eight-shaped piece of gauze without applying tension around the screw wounds. If the fabric has loose filaments, they should be bent inwards to prevent them from getting in the wound. Using his/her fingers, the patient will press the gauze against the skin and secure it to keep a firm pressure, limiting the movement of the skin around the pins. After the first day, and once the wounds have dried, we do not recommend covering them with a piece of gauze, and it is preferably to let them free.
- The complete fixator must be cleaned using larger gauzes.
- After cleaning is finished, all the material used must be thrown away and the patient must wash his/her hands again.
- After 10 days of the implantation, and if authorised by the surgeon, the patient can have a shower with the fixator and use standard soap and water for cleaning. But the screw cleaning protocol will remain unchanged throughout the treatment.
- The symptoms that might indicate the presence of an infection are:
  - Redness around the insertion point of the pin.
  - Suppuration of the screw wound.
  - Dense secretion from the half-pin wound.
  - Mobility or loosening of the screw.
  - Persistent pain in the area of the screw.

## Conclusions

Bone elongation through callotasis is a relatively simple procedure from a surgical point of view. However, the large number of variables to consider when planning this type of treatment and their duration, which make them susceptible to many different complications, make it advisable to leave these cases in the hands of experts who know the obstacles they may find.

The existence of reference centres is a great advantage when it comes to putting these patients in the hands of surgeons used to dealing with such cases, but they are also an excellent opportunity for less experienced doctors to be trained in order to improve the mastering of these techniques.

Bone elongation results are amazing and highly beneficial to the patients’ life quality. But we should give this type of surgery its right value and we must plan and face it in the most responsible way. Our task is to restore the anatomy of the locomotor system mechanically and, which is even more important, biologically, since the whole future life of these bones and joints will depend on it.

A good use of the external fixation—in particular and in this case, the monolateral one—leads us to meet various orthopaedic and surgical techniques which were unthinkable years ago and which nowadays are a standard practice in paediatric and adult orthopaedics units worldwide. Circular external fixation is also very useful, and the author uses it when necessary. Indications for circular frames are clear when lengthening is associated with complex angular or rotational deformities. In simple tibial lengthenings, those kind of fixators can limit axial deviations in cases of dysplasia or when a deformity is present. Thus, the elongation of bones and their soft tissues (distraction osteogenesis and histogenesis) through external fixation is a method of treatment for various diseases that generates good results although it has difficulties. This guide aims to smooth the path for those who start using this technique, which seems simple but is highly complex in its background.
